# A Quantitative Sensory Testing Approach to Pain in Autism Spectrum Disorders

**DOI:** 10.1007/s10803-019-03918-0

**Published:** 2019-02-15

**Authors:** Sarah Vaughan, Francis McGlone, Helen Poole, David J. Moore

**Affiliations:** 1 School of Natural Sciences and Psychology, Psychology Department, Liverpool John Moores University, Liverpool, L3 3AF UK; 2 Faculty of Social Sciences, School of Psychology, Chester University, Chester, CH1 4BJ UK; 3 Institute of Psychology, Health and Society, University of Liverpool, Liverpool, L69 3GL UK; 4 Department of Natural Sciences and Psychology, Liverpool John Moores University, Liverpool, L3 3AF UK

**Keywords:** Autism, Quantitative sensory testing, Pain, Somatosensation

## Abstract

Sensory abnormalities in autism has been noted clinically, with pain insensitivity as a specified diagnostic criterion. However, there is limited research using psychophysically robust techniques. Thirteen adults with ASD and 13 matched controls completed an established quantitative sensory testing (QST) battery, supplemented with measures of pain tolerance and central modulation. The ASD group showed higher thresholds for light touch detection and mechanical pain. Notably, the ASD group had a greater range of extreme scores (the number of z-scores outside of the 95% CI > 2), dynamic mechanical allodynia and paradoxical heat sensation; phenomena not typically seen in neurotypical individuals. These data support the need for research examining central mechanisms for pain in ASD and greater consideration of individual difference.

**Electronic supplementary material:**

The online version of this article (10.1007/s10803-019-03918-0) contains supplementary material, which is available to authorized users.

## Introduction

In addition to the most striking lifelong effects of impaired communication, socialization and restrictive/repetitive behaviours in autism spectrum disorder (ASD), there is a high prevalence of sensory perceptual anomalies (Baranek 2002). Evidence for which has relied on autobiographical, observational or behavioural measures (Moore 2015) which has demonstrated, amongst an array of sensory disturbances, an absence of typical pain behaviours (e.g. absence of hand withdrawal reflex or a lack of protective body positioning) when encountering pain (Bursch et al. 2004; Gillberg and Coleman 2000; Mahler 1952; Rothenberg 1960; Wing 1996). There is further evidence that autistic individuals have aversions to touch (Grandin 1992, 1995; Williams 2015), suggesting that light tactile sensation might be a source of discomfort, indicating a potential hypersensitivity to tactile stimuli (Kaiser et al. 2016; Moore 2015). However, such methods are typically not generalizable because it is unclear whether the case investigated is representative of the wider body of “similar” instances. Further validation of this phenomenon is given by the re-incorporation of sensory responses as a feature in diagnostic texts suggesting that it is a central clinical finding in autism (APA 2013). There is however, a dearth of rigorous psychophysical experimental evidence to support these claims. Therefore, the current study aims to clarify the characteristics of *pain* sensitivity associated with ASD using a psychophysically robust experimental case-control design.

*Pain* is multifaceted, defined as a distressing experience associated with actual or potential tissue damage; with sensory, emotional, cognitive and social components (IASP 2012; Williams and Craig 2016). Together, the percept, and the subjective reaction act as a warning system so that individuals learn to avoid dangerous stimuli (Yasuda et al. 2016), whilst also promoting behavioural analgesia (Eccleston and Crombez 1999). A disruption to this system could result in a lack of these learned behaviours.

Potentially nociceptive (painful) stimuli are detected by specific somatosensory receptor neurons (nerve fibres), known as nociceptors which can be classified into three different types: Aβ, Aδ and C-fibre (Besson 1999; Delmas et al. 2011; Djouhri and Lawson 2004; Lumpkin and Caterina 2007). Nociceptive messages are typically mediated by Aδ, and C-fibres, the functionality of which, in neurotypical populations, has been well described (for reviews see Basbaum et al. 2009; Basbaum and Jessell 2000; McGlone and Reilly 2010; Meyer et al. 2008). Before these signals generate a perception of ‘pain’ they are centrally integrated in the dorsal horn of the spinal cord and transmitted to the brain via the spinothalamic tract (Basbaum and Jessell 2000; Iggo 1977; Nafe 1934; Schiller 1956). The final process in the pain experience is the social communication of pain which can be observed in stereotyped pain behaviours (Craig 2015) and self-report – and which is neither simply, nor directly, associated with the level of nociceptor activity; nociceptor activity can produce more or less pain depending on a range of factors (Loeser 2012). De-coding whether these underlying mechanisms are altered in autistic individuals will give insight into the pain behaviours observed in this population.

Recently a few studies have begun to disentangle the underlying sensory mechanisms of somatosensory dysfunctions in ASD using psychophysical methods, the earliest of which focused on tactile sensitivity, investigating this with vibrotactile stimuli (Blakemore et al. 2006; Cascio et al. 2008; Guclu et al. 2007). Blakemore et al. (2006) reported a frequency dependent hypersensitivity in adults with Asperger’s compared to neurotypical controls. Conversely, Guclu et al. (2007) and Cascio et al. (2008) report no significant difference between the vibrotactile thresholds of children and adults with ASD and controls, suggesting that effects may be a result of specific frequencies, sites or other methodological differences.

Regarding pain perception, the focus has generally been towards thermal testing, with similarly mixed findings. Thermal pain hypersensitivity but normal thermal detection has been reported in adults with ASD (Cascio et al. 2008). Adolescents are reported to have the inverse results; normal thermal pain thresholds, but a hyposensitivity to innocuous thermal stimuli (Duerden et al. 2015). No differences in thermal detection thresholds and electrical pain were observed by Yasuda et al. (2016) and Bird et al. (2010), however, pressure pain thresholds were lower in autistic individuals compared to controls (Fan et al. 2014). This pattern of findings suggests no systematic change in psychophysically determined pain thresholds for autistic individuals compared to controls. This is not to suggest that pain response in ASD is typical, both Fründt et al. (2017) and Duerden et al. (2015) report paradoxical heat sensations, a phenomenon where gentle cooling is perceived as hot or burning (Magerl and Klein 2006), in several of their autistic participants. This phenomenon usually does not occur in healthy individuals.

Considering the paucity of evidence paired with the mixed results, probably due to the heterogeneity of participants (e.g. autism symptom severity or comorbidities) and differences regarding methods and sub-modalities investigated, the disentanglement of the underlying mechanisms of somatosensory dysfunctions in ASD is limited and there is no gold standard on how these features should be assessed in ASD.

Several recent investigations (Blakemore et al. 2006; Cascio et al. 2008; Duerden et al. 2015) have utilised methodologies that have been collated into the standardised quantitative sensory testing battery developed by The German Research Network on Neuropathic Pain (DFNS; Rolke et al. 2006). This method allows for the quantification of clinically significant perception and pain thresholds (Werner et al. 2013) assessing the function of small and large diameter nerve fibres (Hansson et al. 2007). If used in its entirety this method allows researchers to assess nerve function across the full range of modalities; vibration, pressure, thermal, and mechanical (Moloney et al. 2012) in a standardised manner. The focus on a single or a limited number of these sub-modalities limits previous studies. One study, however, has utilised this full battery, therefore, providing the most comprehensive assessment of somatosensory function in ASD to date (Fründt et al. 2017). More extreme somatosensory responses (i.e. hyper- or hypo-sensitivity) or somatosensory phenomena not typically observed in those without neuropathy (i.e. dynamic mechanical allodynia or paradoxical heat sensations) were observed in the ASD group, however, there were no group differences reported for global or systemic changes in somatosensory function.

This study will similarly employ the standardised battery, conducting an independent replication of Fründt et al. (2017) and utilise the published normative reference values (Rolke et al. 2006) as they provide a determinant of sensory loss and gain that supersedes the standard group differences analysis - meaning clinically significant sensitivities in ASD can be determined. Furthermore, this battery has been extended to include a measure of pain tolerance and central pain processes, utilising the cold pressor test (von Baeyer et al. 2005) and Conditioned Pain Modulation (CPM; Yarnitsky et al. 2015), respectively. Including tolerance allows a wider range of psychophysics to be measured; threshold (the minimum intensity of a stimulus that is perceived as painful), suprathreshold (increases the frequency of nociceptive messages) to tolerance (the maximum intensity of a pain-producing stimulus that a subject is willing to accept in a given situation (Chapman et al. 1985; IASP 2012). Tolerance also includes additional components such as pain motivation; to quantify said motivation; self-reported desires to avoid pain were measured. CPM represents one type of central pain process; that of descending spinal modulation, that although not currently tested in ASD populations, is a paradigm easily implemented in a laboratory setting. It is a process whereby one noxious stimulus inhibits the perception of a second noxious stimulus, where greater reductions in pain are thought to reflect greater pain inhibitory capacity (Martel et al. 2013; Nir and Yarnitsky 2015). The addition of each will give insight into tolerance, pain motivation, and central pain processes in ASD.

## Methods

### Participants

Twenty-six adults (14 males) covering an age range between 18 and 52 years were recruited (*M* = 27.15, *SD* = 8.50) to this case-control study. ASD participants were recruited from a specialist diagnostic service within a local hospital trust and had received a diagnosis based on the DISCO (Diagnostic Interview for Social and Communication Disorders) and/or ADOS (Autism Diagnostic Observation Schedule) from a trained clinician. Diagnosis letters were obtained from participants where possible, which confirmed diagnosis and IQ values > 70. Those suffering from chronic pain, eczema, epilepsy or asthma were excluded. Additionally, any with a reported history of a psychiatric disorder or learning disability were excluded. Thus, 13 ASD participants were included in the study; there were seven males and six females with a mean age of 27.22 years (SD = 9.19). No participant reported any medication use for depression or anxiety, although one reported the use of Amlodipine (for angina) and one reported the use of Lansoprazole (for ulcers).

Thirteen control participants without a diagnosis of ASD were recruited through advertisement, selected to match each autistic individual on age (*M* = 27.08, SD = 8.129) and gender (7 males). All were subject to the same exclusion/inclusion criteria above. Although not explicitly matched on IQ, the control group were from the general population, suggesting IQ > 70. All participants in both groups were without pain medication or alcohol at least 24 h before the investigation.

As groups (n = 13 per group) were age and gender matched they did not significantly differ; *t*(22) = − 0.045, *p* = .964 and χ^2^(1) = 0, *p* = .652, respectively. As expected groups had significantly different AQ score (autism quotient: (Baron-Cohen, Wheelwright, Skinner et al. 2001) scores, *t*(24) = − 6.003, *p* = .000, with the ASD group scoring higher (see Table 1 below for descriptive statistics).

**Table 1:** Characteristics and questionnaire results of ASD and control group

Characteristic	ASD	Controls	Total
No. of participants	13	13	26
No. of participants with: ASC	1	–	1
HF autism	2	–	2
Asperger’s	10	–	10
Age	27.22 ± SD 9.19	27.08 ± SD 8.13	27.15 ± SD 8.50
Gender			
Female	6	6	12
Male	7	7	14
Autism quotient (AQ)	32.00 ± SD 6.58	15.38 ± SD 7.50	23.69 ± SD 10.94

*HF* high functioning and *ASC* autism spectrum condition

All values are given as mean ± SD

*p < .05

The study was approved by Liverpool John Moores Ethics Committee (REC Ref: 15/NSP/023) and NHS Health Research Authority ethics committee (Ref: 16/EM/0402) and all participants gave written informed consent.

## Procedure and Design

To quantify self-reported autistic trait severity participants completed the AQ (Baron-Cohen et al. 2001). QST was performed first. This standardized battery provides a sensory profile that consists of 13 parameters (see Table 2 below, Rolke et al. (2006) and supplementary methods for full description). Additional cold pressor and CPM tests were added to the battery and all tests were performed in the same order, using the same set of standardised instructions and performed on the same site on each participant.

**Table 2:** Details of standardised quantitative sensory testing battery, tests and associated peripheral sensory channel

Group no.	Description	Test (abbreviation)	Peripheral sensory channel
1	Thermal detection thresholds for the perception of cold, warm and paradoxical heat sensations	Cold detection threshold (CDT)	A-delta
		Warm detection threshold (WDT)	C
	Performed using a medoc pathway stimulator, ramped stimuli 1°C/s, baseline temperature 32 °C and a 9 cm² thermode	Paradoxical heat sensations (PHS)	C, A-delta
		Thermal sensory lumen (TSL)	C, A-delta
2	Thermal pain thresholds for cold and hot stimuli (as above)	Cold pain threshold (CPT)	C, A-delta
		Heat pain threshold (HPT)	C, A-delta
3	Mechanical detection thresholds for touch and vibration	Mechanical detection threshold (MDT)	A-beta
	Performed using a modified set of von Frey hairs (0.25–512 mN) with five ascending and five descending stimulus intensities and a 64 Hz tuning fork (8/8)	Vibration detection threshold (VDT)	A-beta
4	Mechanical pain sensitivity, including thresholds for pinprick, stimulus–response functions for pinprick sensitivity, dynamic mechanical allodynia and pain summation to repetitive pinprick stimuli	Mechanical pain threshold (MPT)	C, A-delta
		Mechanical pain sensitivity (MPS)	C, A-delta
	Performed using a set of weighted pin-pricks that exert forces of 8, 16, 32, 64, 128, 256 and 512 mN	Dynamic mechanical allodynia (DMA)	C, A-delta
		Wind-up ratio (WUR)	C, A-delta
5	Pressure pain threshold	Pressure pain threshold (PPT)	C, A-delta
	Performed using an algometer with a 1cm² probe area, where stimulus intensity is gradually increased at a ramp rate of 50 kPa.s		
6	*Cold pain threshold and tolerance*	*Cold pressor test*	*C, A-delta*
	*Performed with a custom cold pressor which maintains water at 2 °C, participants submerge their dominant hand in the water stating “pain” for threshold and tolerance is measured as the point at which the hand is voluntarily removed*		
7	*Pain modulation*	*Conditioned pain modulation (CPM)*^a^	–
	*Performed using an algometer with a 1 cm²-probe area, where stimulus intensity is gradually increased at a ramp rate of 50 kPa/s and a cold pressor test (see 6.)*		

Test order: Cold and warm thermal detection thresholds are acquired first followed by paradoxical heat sensations during thermal sensory lumen of alternating warm and cold stimuli (no. 1). Cold and heat thermal pain thresholds (no. 2) are then determined. Then follows; mechanical detection (no. 3), mechanical pain (no. 4), stimulus/response functions with dynamic mechanical allodynia (no. 4), wind-up ratio (no. 4), vibration (no. 3), pressure pain (no. 5), cold pressor test (no. 6) and lastly conditioned pain modulation (no. 7) is performed

Additional tests that are not part of the DFNS QST battery (i.e. no. 6 & 7) are given in italics

^a^This is a measure of central pain processes not of the peripheral sensory channels; although these channels are involved in the initial detection of the relevant stimuli (see no. 4 and 5)

### Cold Pressor Test

A custom cold pressor (Dancer Design), which maintains water in a stimulus tank at a predefined temperature (2 °C), measure both cold pain tolerance and threshold. A control unit containing a temperature controller drives water taken from a reservoir of ice water (maintained at 0 °C) through the stimulus tank at a controlled rate, therefore, maintaining the requested temperature within 0.10 °C (see supplementary materials for full description and schematic diagram).

Pain threshold is defined as the elapsed time between arm immersion and the first report of a pain sensation. Pain tolerance is defined as the elapsed time until the hand is voluntarily removed. Since the Cold Pressor test induces pronounced sympathetic activation and vasoconstriction, the maximum duration of limb immersion was set at 3 min (Mitchell et al. 2004).

### Conditioned Pain Modulation (CPM)

To assess CPM baseline pressure pain thresholds (PPT) was firstly performed on the right upper trapezius, approximately 2 cm from the acromioclavicular joint with a handheld pressure algometer (Somedic) with a 1 cm^2^ probe area. The threshold was determined with an ascending stimulus intensity, applied as a slowly increasing ramp of 50 kPa/s until participants report a painful sensation. Immediately following the assessment of PPT, participants underwent a cold pressor test, immersing their hand up to the wrist in a stimulus tank of 2 °C water. Twenty seconds following hand immersion, PPT was re-assessed on the right trapezius (i.e. the same site as baseline assessment).

### Avoidance and Motivation Scores for Pinprick Stimuli Including Stimulus/Response Function (MPS/DMA)

Pain experience is more than just the sensory experience, the functional purpose of pain is to create a motivational state to avoid future harm (Eccleston and Crombez 1999). To measure the motivation to avoid experiencing painful stimuli, participants were asked that, for every stimulus that was given a pain rating (a value above 0 on a numeric rating scale of 0–100 where 0 means no pain and 100 means the most intense pain imaginable, any figure over 0 is considered to be a rating of pain: see the QST supplementary materials for MPS, DMA and WUR) during mechanical pain sensation (MPS), dynamic mechanical allodynia (DMA) and wind-up ratio (WUR), to rate how much they would like to avoid feeling that stimulus. Avoidance was rated using the same scale as the aforementioned QST parameters of 0–100; 100 being “would never like to experience the stimulus again”. MPS avoidance was calculated as the geometric mean of all avoidance ratings for pinprick stimuli, while DMA avoidance was the geometric mean of all avoidance ratings corresponding to the dynamic stimuli. The wind-up ratio avoidance was calculated as the ratio of the mean of the five series avoidance ratings divided by the mean of the five single stimuli avoidance ratings.

## Data Preparation

### QST

Preparation of individual participants data followed the guidance of the DNFS (Rolke et al. (2006). For pinprick (MPS/DMA), as well as their corresponding avoidance measures, a small constant (+ 0.1) was added prior to log-transformation to avoid a loss of zero rating values (Bartlett 1947; Magerl et al. 1998).

For each individuals raw scores it has been previously established that all QST data except Paradoxical Heat Sensations (PHS), Cold Pain Threshold (CPT), Heat Pain Threshold (HPT), and Vibration Detection Threshold (VDT) follow either a logarithmic progression (i.e. stimulus intensity of the pin prick stimuli are 8 mN, 16 mN, 32 mN, …) or that these data always conform to this distribution, therefore individual participants raw scores were logarithmically transformed before creation of mean values for analysis (Magerl et al. 2010; Rolke et al. 2006). To permit normalisation for age, gender and testing site, each individual’s QST data were z-transformed by subtracting the mean value of the corresponding published QST reference value followed by a division by the respective standard deviation from the normative database for the appropriate age and gender group; for each QST parameter using the following expression:

$${\text{Z}} - {\text{score }}={\text{ }}\left( {{{\text{X}}_{{\text{single}}}}_{{{\text{participant}}}}-{\text{ Mea}}{{\text{n}}_{{\text{norms}}}}} \right)/{\text{S}}{{\text{D}}_{{\text{norms}}}};$$

An additional reason for this transformation is that it results in a QST profile where all parameters are presented as standard normal distributions. For clarity and ease, in order to think in terms of gain (lower thresholds or lower intensity stimulus required for detection or pain report) or loss of function (higher thresholds, or greater intensity required for detection or pain report), the algebraic sign of Z-score values was adjusted so that it would reflect a participant’s sensitivity to this parameter. Z-values above “0” indicate a gain of function, when the patient is more sensitive to the tested stimuli, while a score below “0” indicate a loss of function referring to a lower sensitivity. Thus all required reversing, with the exception of CPT, MPS, DMA and WUR. For PHS and DMA it is a priori impossible to assess a pathological reduction since these signs are normally absent in a healthy population. If the resulting Z score exceeds 1.96, it is outside the 95% confidence interval of the standard normal distribution with zero mean and unit variance, independent of the original units of measurement. An advantage beyond that of establishing whether any participant, neurotypical or ASD, has clinically significant sensory loss or gain, is that of placing all the data into a standardised space where individuals QST patterns can be explored. This somewhat allows us to navigate the ASD phenotype and look at individual level data.

QST data were re-transformed and raw values are presented in Table 3 as mean ± SD to ease understanding, and so that data could be presented in terms of the individual units of measurement e.g. temperature in ˚C. All inferential statistics for QST were conducted on z-scored data. Where values are presented as z-scores figures and tables state this. All statistical calculations were performed with SPSS.

**Table 3:** Untransformed data values of QST test parameters given for each group

Parameter (Mean ± SD)	ASD	Controls	p value	Effect size
QST parameter				
CDT (˚C)	30.423 ± SD 0.661	30.503 ± SD 1.019	0.579	δ = 0.2
WDT (˚C)	34.618 ± SD 1.545	34.092 ± SD 0.758	0.287	δ = 0.5
TSL (˚C)	5.103 ± SD 2.415	4.550 ± SD 1.951	0.515	δ = 0.2
PHS (n)	0.150 ± SD 0.555		.317^b^	δ = 0.1
CPT (˚C)	20.615 ± SD 6.651	16.546 ± SD 12.021	0.491	δ = 0.3
HPT (˚C)	42.297 ± SD 3.576	40.918 ± SD 2.598	0.272	δ = 0.4
MDT (mN)^a,^*	8.238 ± SD 7.638	3.267 ± SD 2.564	0.005	δ = 1.2
MPT (mN)^a,^*	125.296 ± SD 157.378	46.687 ± SD 37.438	0.007	δ = 1.2
MPS (PR)+	1.860 ± SD 2.382	2.048 ± SD 2.570	0.685	δ = 0.2
DMA (PR)+	0.863 ± SD 2.698		0.379^†^	δ = 0.4
WUR (PR)+	5.498 ± SD 7.533	2.021 ± SD 2.369	0.203	δ = 0.5
VDT (/8)	7.282 ± SD 0.880	7.744 ± SD 0.512	0.129	δ = 0.8
PPT (kPa)+	307.205 ± SD 60.124	361.846 ± SD 105.572	0.162	δ = 0.6
Additional sensory tests (mean ± SD)				
CP threshold (s)	12.245 ± SD 7.901	11.284 ± SD 8.891	0.773	δ = 0.1
CP tolerance (s)	37.278 ± SD 45.493	28.235 ± SD 17.873	0.511	δ = 0.3
CPM1 (kPa)	317.770 ± SD 111.456	345.000 ± SD 95.076	0.173	See results
CPM2 (kPa)	428.920 ± SD 202.720	393.46 ± SD 123.799	0.173	See results

Group raw data values for each QST parameter and additional sensory tests given as mean ± SD to aid understanding in terms of their actual unit of measurement i.e. temperature in Celsius

All p values and effect sizes given for QST parameters are for the inferential statistics conducted on transformed data as discussed in the methods section

*p < .05

^a^Values are presented as geometric means

^b^Non-parametric Mann–Whitney U conducted for these parameters as they did not meet assumptions, all other parameters met parametric assumptions and therefore independent samples *t*-test conducted

### Additional Sensory Tests

These data did not undergo the same transformation as the QST data. This was to ensure that results were comparable to other published data where possible.

## Results

It was possible to obtain all QST data in all 26 participants. For one-control participant WUR, avoidance scores could not be calculated because the denominator (mean rating for the single stimulus) was zero.

### QST Reference Data Between Groups

**Fig. 1:**
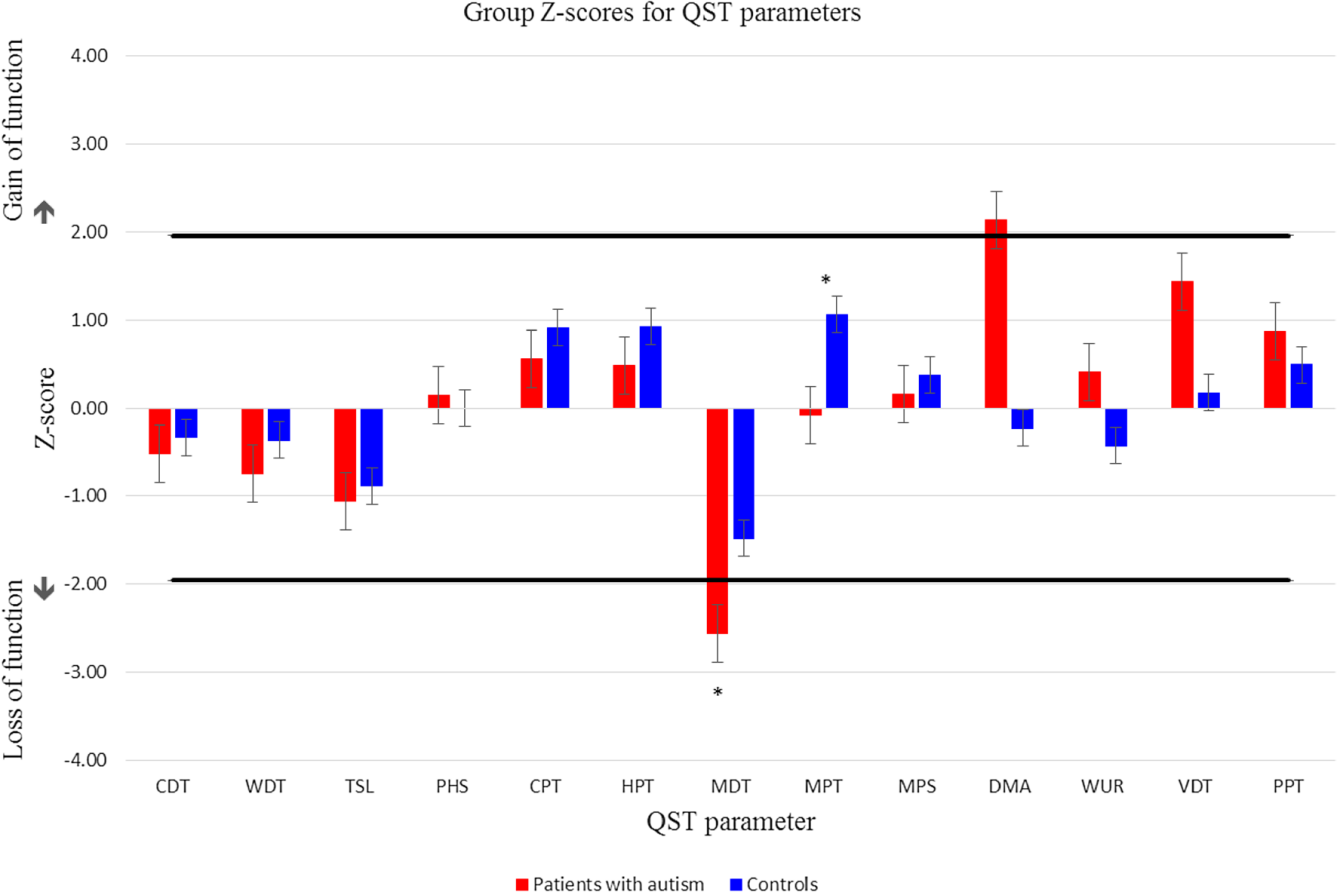
Adjusted Z-score data for ASD versus control group, across all 13 QST parameters including standard error bars. * Indicates significant group differences. Any column that extends outside the 95% confidence interval of the normal distribution of healthy subjects (= area between the black lines) signifies sensory changes

Group comparisons of each QST parameter’s mean Z score, using independent t-tests, revealed a significant difference for mechanical detection and pain threshold (MDT & MPT). The ASD group (*M* = 8.238 mN) required a significantly greater force to detect light touch than the control group (*M* = 3.267), *t*(24) = − 3.073, *p* = .005. They also reported pain at a greater force (*M* = 125.596 mN) for mechanical pain than controls (*M* = 46.687 mN) *t*(24) = − 2.950, *p* = .007. The ASD group shows hyposensitivity to mechanical stimuli compared to controls; although only in the case of MDT does this reflect hypoesthesia for mechanical detection (as shown by a value that falls outside the 95% confidence interval of the published reference data; see Fig. 1).

## Additional Sensory Tests

### Cold Pressor Test

Independent t-tests revealed there were no significant group differences for cold pressor threshold or tolerance, *t*(24) = − 0.291, *p* = .773 and *t*(24) = − 0.667, *p* = .511, respectively (see Table 3 for mean values).

### Conditioned Pain Modulation

**Fig. 2:**
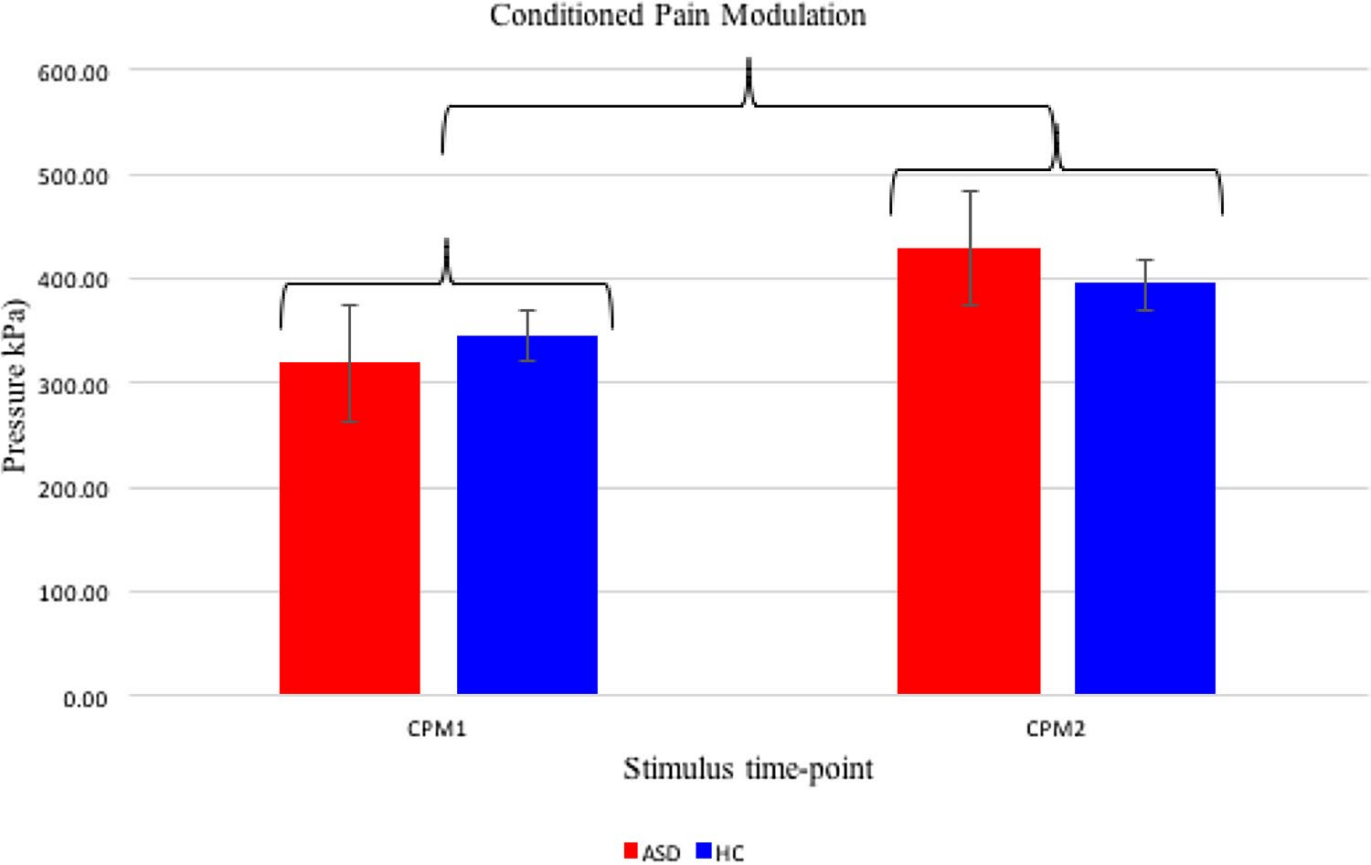
Group data for conditioned pain modulation, including standard error bars, given as raw data values. * Indicates significant stimulus time-point differences

A repeated measures ANOVA revealed that pressure pain was significantly modulated by a cold pressor test *F*(1) = 12.793, *p* = .002, *r* = 0.6, as the pressure pain threshold increased after the hand was submerged for the 20 s, across groups, supporting the existence of a CPM effect in the sample. The magnitude of this CPM effect, however, did not significantly differ between groups, *F*(1) = 1.974, *p* = .173, *r* = 0.2 (see Fig. 2). Cold pressor pain mediated pressure pain, as shown by the increase in pressure required to elicit a pain response regardless of group (see Table 3 for mean values and Fig. 2 for illustration).

### Avoidance Scores for Pinprick Stimuli Including Stimulus/Response Function (MPS/DMA)

For avoidance scores, *t*-tests were only conducted when parametric assumptions were met; otherwise, Mann–Whitney U test was used. There were no group differences for MPS avoidance (*t*(24) = − 0.260, *p* = .797). Neither, DMA nor WUR avoidance differed between groups (*U* = 68.000, z = − 0.879, *p* = .194 and *U* = 66.000, z = − 0.958, *p* = .178).

### QST Profiles of z-Transformed Data in Individual Participants

**Fig. 3:**
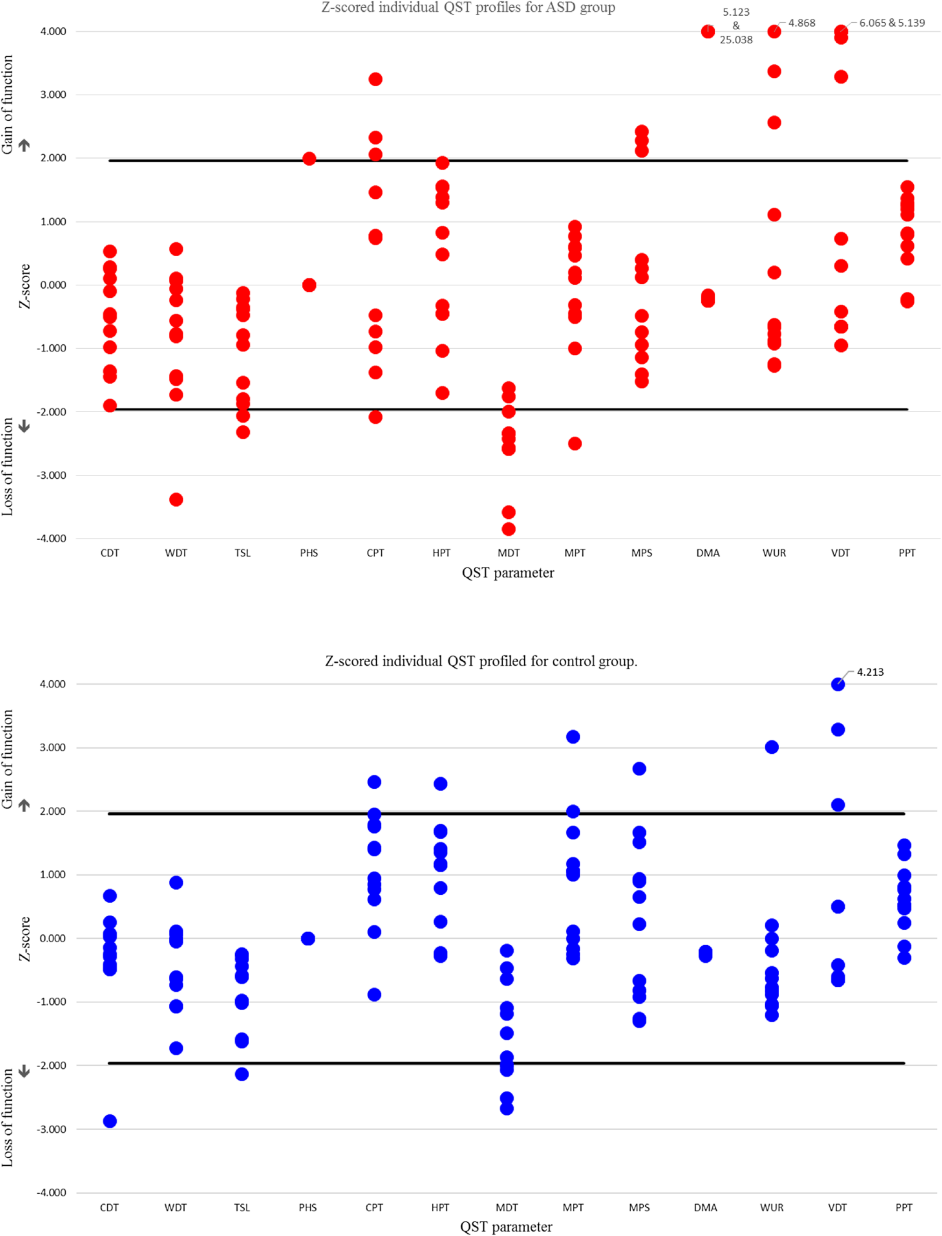
Individual results of QST parameters given as Z-scores of autism participants (red) versus controls (blue). Any marker that extends outside the 95% confidence interval of the normal distribution of healthy subjects (= area between the black lines) signifies sensory changes. Values that extended beyond four standard deviations were given a maximum value of 3.999 or − 3.999 and true values are given next to the marker. Data were constrained in this way to ensure that figures could be clearly interpreted

Overall, there were a greater number of z-scores (see Fig. 3) that fell outside of the 95% confidence levels within the total sample than would be expected by chance (n = 48, allocated to 19 individuals). For a sample of this size, with 13 QST parameters, 95% confidence interval (CI) levels estimate that 15 values would lie outside the 95% CI level of the DFNS reference data. This variance is driven by the larger number of abnormal z-scores in the ASD group (n = 32 allocated to all 13 individuals) compared to controls (n = 16 allocated to six individuals); who show typical numbers of outlying scores.

Intra-individually, 95% CI dictates that one z-score in the 13 QST parameters would potentially be outside this level, which suggests that only 15 of our participants are showing atypical QST patterns (where the number of z-scores outside the 95% CI ≥ 2). A greater number of ASD individuals were found to have extreme scores compared to controls, and the range of these scores was wider in ASD individuals (2–5) compared to controls (2–3). However, the average number of these scores per participant, in those that showed this atypical pattern, was similar between the groups (see Table 4 below for descriptive statistics). Therefore, although a greater percentage of individuals with ASD may show atypical patterns of pain response, when considering these altered responses they may be within a range seen in a similar neurotypical group.

**Table 4:** Number of participants with atypical QST patterns and the mean number of abnormal z-scores of each participant

	ASD	Controls	Total
No. of participants	10	5	15
Abnormal z-scores	2.9 ± SD 1.101	2.8 ± SD 1.366	2.867 ± SD 1.325
Range of abnormal z-scores	2–5	2–3	2–5

Total number of participants in each group showing abnormal values (where the number of abnormal values ≥ 2; i.e. are outside the 95% CI of the reference data)

The number of abnormal values per individual in the groups is given as a mean ± SD, and range

Furthermore, 1 autistic individual showed sensory distinctive features in the form of PHS; experiencing a warm, hot or painfully hot sensation in response to the cold stimulation, and two felt allodynia to non-painful stimuli (DMA), that usually does not occur in healthy subjects. These observations suggest that in this small population of individuals with ASD there are notable changes in peripheral function. Although these features do not appear to be typical of ASD, this does suggest sub-groups of ASD in which altered somatosensory processing may be present. Further, it appears that differences in sensory processing in some individuals may not simply be in terms of magnitude of response. Rather, it might reflect the presence of phenomena not typically seen in neurotypical individuals.

## Discussion

The current study investigated somatosensory perception in autistic individuals to test the hypothesis that the different pain behaviours observed in anecdotal accounts were the result of an alteration in somatosensory mechanisms. For this reason, and to allow the comparison to published norms, 13 autistic adults and 13 age- and gender- matched control participants without autism, underwent a standardised and normed QST protocol (DFNS: Rolke et al. 2006). No observable consistent pathological QST pattern suggesting a defined nerve fibre dysfunction, which could account for the altered pain behaviours observed, was found. The ASD group showed no systematic changes in their QST pattern.

Group differences were found, however, for both mechanical pain threshold (MPT; pinprick stimuli) and mechanical detection threshold (MDT; von Frey filaments), with the ASD group showing higher thresholds for both. Although the ASD group had higher thresholds compared to the control group, data for both groups reside within the normal distribution of healthy individuals, as established by the DFNS, indicating that although the ASD group may be less sensitive to mechanical pain than controls this sensitivity is not clinically significant. However, ASD group mean value for MDT fell outside the normative range for healthy individuals, suggesting a clinically significant degree of sensory loss at the group level. Normal z scores for other clinically related QST parameters—such as vibration detection threshold—do suggest, however, typical Aβ-fibre function (Grone et al. 2012).

Vibrotactile and punctate stimulation are both communicated via Aβ-fibres, though detected by different receptor pathways, which may account for the aforementioned differences. High frequency vibration is detected via rapidly adapting Pacinian corpuscle and generally have a large receptive field. Mechanical stimulation, on the other hand, are detected via slowly adapting Merkel cell-neurite complex receptors and is tactile detection via indentation depth (Delmas et al. 2011). Different Aβ-fibre phenotypic alterations may therefore be present and be stimuli specific, due to detection of such stimuli by their specific receptors. Such differences are highlighted in the evidence when contrary to the sensory loss of MDT measured by von Frey, increased sensitivity to vibration is reported (Cascio et al. 2008). There is greater difficulty in comparing vibration results in the literature, due to the varied vibration frequencies used (Blakemore et al. 2006; Guclu et al. 2007), yielding very different results which may similarly be a result of different receptor activation (Lumpkin et al. 2010; McGlone and Reilly 2010; McGlone et al. 2014). It must also be noted that the use of a tuning fork for vibrotactile assessment is sensitive enough to identify neuropathy—as intended—however, may not be sensitive enough to measure more subtle changes in threshold. Findings for MDT are in line with Fründt et al. (2017) who similarly report a significant loss of function for mechanical detection in ASD participants using the same standardised testing from the QST battery.

Similar to Fründt et al. (2017) who report PHS and DMA in two autistic individuals (see also Duerden et al. 2015), three participants showed distinctive sensory features in the form of paradoxical heat sensations (n = 1; PHS) and dynamic mechanical allodynia (n = 2; DMA), that do not usually occur in healthy individuals on the upper limbs (Rolke et al. 2006) and were not observed in the control group. Given that the different QST parameters did not reveal any specific signs of nerve fibre dysfunction in both studies, we concur with the author’s suggestion that central mechanisms determine PHS in the ASD groups. Studies of patients with CNS demyelination confirm central processing issues that result in PHS (Hansen et al. 1996). Limited research has attempted to understand the central processing of pain in ASD using neuroimaging techniques. This research supports the idea that changes in pain processing in ASD is complex: suggesting that there is an initial processing which is similar to controls, however, there is a reduction in neural activity during sustained pain that is not present in controls (Failla et al. 2017). This gives further support to the need to be flexible about how pain experience is considered in ASD.

A further phenomenon, observed by this study and that of Fründt et al. (2017) is that of DMA. Both studies are the first to experimentally measure DMA in ASD, observing this in a subset of the ASD groups. DMA is the experience of perceiving innocuous touch, such as gentle stroking, as aversive, a phenomenon observed in ASD sensory over-responsivity literature (Baranek and Berkson 1994; Green et al. 2015; Reynolds and Lane 2008). Central sensitisation i.e. changes in signalling in the spinal cord (Campbell and Meyer 2006), is commonly thought to underlie DMA (Gierthmühlen et al. 2012), as it is the increased response of neurons to stroking stimuli. Intriguingly, some groups have offered a peripheral explanation for DMA (Liljencrantz et al. 2013), whereby an alteration in C-tactile afferent function, which typically mediates a pleasant percept associated with low force slow stroking touch, communicates noxious experience. This explanation then lends weight to research suggesting that an early mechanism behind ASD may be an alteration in CT fibre function (Cascio et al. 2018; Gordon et al. 2013; Kaiser et al. 2016; Walker and McGlone 2013). It is clear that this proposition requires further investigation. However, QST cannot fully distinguish between central and peripheral alterations (Mücke et al. 2014), therefore we can only speculate at this time.

There are striking similarities between our findings and those of Fründt et al. (2017). Both were independently conducted, in parallel, and sought to use the DFNS QST protocol to identify differences that might exists in somatosensory function is ASD. Both studies found little evidence for a diagnosis-wide change in either somatosensory detection or pain thresholds. Both also found that when Z-scores were compared to published norms more autistic individuals showed a-typical data points, suggesting that individual differences may be of importance. This replication is particularly powerful as psychological sciences wrestle with the reproducibility crisis (Aarts et al. 2015). Here, independent verification of findings has been achieved, to provide a platform upon which to build future research.

An advantage of the standardised QST method is the published normative data which provides clear definitions of sensory loss and gain. The ASD phenotype can drastically differ and has large individual differences meaning the typical group analyses may not be advantageous to understanding this spectrum condition. Such published norms, which an individual’s QST pattern can be compared to, provides the opportunity to quantify individual cases. Individual analyses revealed a greater inter-individual variance with more Z-scores outside the 95% confidence interval of the DFNS published normative values in the ASD group (n = 32). This variance was present in all QST parameters and was not driven by a single participant (n = 13 participants). This might reflect the general heterogeneity of the ASD group; such heterogeneity belies the attempt to group this population under one diagnostic umbrella. The utility of this type of analysis is best shown in Fig. 3, which illustrates the sensory profiles of autistic individuals, and their sensory changes (see results section). This also allows individual differences in the phenotypic presentation of ASD to be considered alongside their QST pattern.

In order to gain a self-report measure of motivation for pain avoidance, individuals were asked: “how much would they like to avoid feeling the stimulus again?”. However, these results were inconclusive. Self-report measures of pain motivation do not appear therefore, to access motivation in a way that provides a clearer or deeper understanding. For this reason, elegant experimental paradigms that have been used in healthy populations for understanding goal attenuation of avoidance behaviour could be adopted and utilised in an ASD population (Claes et al. 2015, 2014; Meulders and Vlaeyen 2012, 2013). Such experiments can implicitly test motivation that goes beyond conscious self-reporting by measuring behavioural responses and understanding avoidance in the context of multiple goals. This could be of vital importance in a population driven to achieve their repetitive or restrictive behaviour patterns regardless of other incoming behaviourally motivational stimuli, such as pain. Furthermore, given that the QST battery revealed typical nerve fibre function and that CPM appeared typical, this approach may help to pull apart the altered pain behaviours by considering top down modulation of pain.

Given the nature of sensory testing- applying a stimulus and recording verbally the perception of that stimulus, the underlying mechanisms can only be judiciously speculated upon. The pain experience in such studies is delivered in controlled environments, devoid of motivational context or other environmental cues. This absence of environmental context, results in a lack of knowledge about how distraction and other psychological effects might affect pain perception in ASD or how they modulate the more simple sensory experience of an input. It is also understandable, brief and cutaneous in nature, which may not reflect the diversity of pain in the real world (the relative merits and challenges of QST measures have been considered extensively elsewhere e.g. Backonja et al. 2013; Rolke et al. 2006). By comparison, naturally occurring pain is frequently endogenous, of longer duration, can be diffuse, and typically involves multiple pain systems. Further, ethical standards of pain induction that mitigate the threat of pain, fundamentally altering the emotional and motivational significance of pain is arguably a key feature of pain that emerges naturally (Edens and Gil 1995). The cost of such control is the potential lack of relevance to naturally occurring pain (Robertson and Low 2006; Rollman 2005). The methodological challenge is to develop techniques that combine the benefits of laboratory control with the relevance of pain that emerges naturally (Moore et al. 2013).

The findings of the present study should be considered in light of several limitations; notably the small sample size, which is common in the literature (Cascio et al. 2008; Duerden et al. 2015; Fründt et al. 2017; Guclu et al. 2007). Many autistic individuals find novel environments distressing and therefore may be unlikely to participate. Additionally, fear of pain and anxiety may likely reduce participation in experimental pain research (Karos et al. 2018). This paired with an exclusion of those with anxiety and depression, placed further limitations on recruitment numbers. This control, however, gives added validity to the results, as these conditions are known to have effects on pain perception (for review see Goesling et al. 2013; Thompson et al. 2016). Future studies should adopt this singular diagnosis approach and increase sample size, regardless of the difficulties caused by frequent psychiatric comorbidities in this population (Joshi et al. 2013). A related limitation is the inability to examine the effect of individual differences on pain responses, specifically IQ. Although participants had been formally assessed for a diagnosis of ASD and had been assessed for IQ in the normal range by a trained clinician, it was not possible to obtain detailed psychometrics. Further independent testing of IQ within the study, was deemed to be burdensome and in the interests of the well-being of the participant, was excluded from the protocol. Additionally, the addition of an IQ test to an already extensive protocol may have increased stress and therefore resulted in an unrepresentative response to stimuli. It would be beneficial in future studies to find mechanisms to understand key individual differences which might affect pain response in ASD. IQ in particular may be an important factor to consider as it has been shown that thermal pain response may be correlated with IQ, with participant’s with a lower IQ score having higher thresholds (Duerden et al. 2015). It was not possible to test this finding in the current research.

In conclusion, there was no systematic alteration to suggest an underlying dysfunction in the cutaneous somatosensory modalities tested in this study. There was a larger number of outlying z-score values within the ASD group. Further, dynamic mechanical allodynia and paradoxical heat sensations were present in some ASD participants, which is typically only observed in patients with peripheral neuropathy. Central processing and integration of sensory information rather than peripheral perception seems to be a better candidate for further research within ASD. In order to test this theory, future studies should focus on combining QST measurements with neuroimaging to detect probable processing differences. Additionally, studies could use experimental paradigms that test pain motivation to assess top-down modulation as a potential cause of altered pain behaviours in this population.

## Electronic supplementary material

Below is the link to the electronic supplementary material.

Supplementary material 1 (DOCX 15 KB)
