# Supplementary material for: A Quantitative Sensory Testing Approach to Pain in Autism Spectrum Disorders
Source: J Autism Dev Disord. 2019 Feb 15;50(5):1607–20. doi: 10.1007/s10803-019-03918-0 (PMC7211210; doi:10.1007/s10803-019-03918-0)
Supplement: Supplementary file 1 — Supplementary material 1 (DOCX 15 KB) [file 10803_2019_3918_MOESM1_ESM.docx]

**QST Methodology: Test Procedures**

**Thermal detection and pain thresholds and the number of paradoxical heat sensations (CDT, WDT, TSL, CPT and HPT)**

Cold and warm detection thresholds were measured first (CDT, WDT), followed by thermal sensory limen (TSL), a procedure of alternating warm and cold stimuli, during which a measure of paradoxical heat sensations (PHS) were established; a phenomenon where gentle cooling is perceived as hot or burning (Magerl & Klein, 2006). Cold and heat pain thresholds were then determined (CPT, HPT). These tests measure Aδ (A-delta) and C-fibre mediated warmth, heat and cold sensations.

Baseline temperature of the thermode (9cm^2^ contact area) was set to 32°C, with cut off of 50°C and -10°C. All thermal tests were performed using a Medoc Pathway Advanced Thermal Stimulator (ATS). All thresholds were obtained with ramped stimuli (1°C/s) that terminated when the subject pressed a button. For thermal detection thresholds the ramp back to baseline was 1°C/s, while pain thresholds returned to baseline at the maximum device capacity of 5°C/s.

The final threshold for CDT and WDT was a mean value of three difference scores from baseline (for example, [WDT1-32+WDT2-32+WDT3-32]/3). The final threshold for TSL, was a mean of the difference value between the three pairs of temperatures i.e. (TSL1 - TSL2) + (TSL3-TSL4) + (TSL5-TSL6)/3. Both cold and warm pain was a mean value of the three threshold values (for example, [HPT1+HPT2+HPT3]/3). In addition to the TSL, participants were asked about paradoxical heat sensations the number expressed was recorded; that is whether the temperature was felt as cold, warm, hot or burning.

**Mechanical detection threshold (MDT)**

A standardised set of modified von Frey hairs (Opti-hair set, MARSTOCKnervtest) was used to measure mechanical detection threshold (MDT) i.e. touch sensibility mediated by Aβ fibres; by applying hairs to a uniform area of skin with a 1-2s contact time. Each hair has a small epoxy bead on a rounded tip in order to avoid nociceptor activation and exerts forces upon bending, between 0.25 and 512mN, graded by a factor of 2. Using “the method of limits”, five threshold determinations were made, each with a series of ascending and descending stimulus intensities. The final threshold was the geometric mean of these series.

**Mechanical pain threshold (MPT)**

Seven weighted mechanical pinprick stimulators (MRC systems) with fixed stimulus intensities that exert forces of 8, 16, 32, 64, 128, 256 and 512mN were applied to a contact area, with a 2s contact time, in order to measure mechanical (pinprick) sensory functions. Using “the method of limits”; stimulators were applied in an ascending order until the first percept of sharpness was reached, followed by a descending order until the first blunt percept was reached, five threshold determinations were made, each with a series of ascending and descending stimulus intensities. The final threshold was the geometric mean of these series.

**Stimulus/response functions: mechanical pain sensitivity (MPS) for pinprick stimuli and dynamic mechanical allodynia (MPS and DMA)**

To obtain a stimulus-response function for pin prick evoked pain (MPS), the same seven set of pinprick stimulators were used (the heaviest pinprick force was about eight times the mean mechanical pain threshold). Participants were asked to give a pain rating for each stimulus on a 0-100 numerical rating scale; 0 indicating no pain, 100 indicating the most intense pain imaginable. This test detects pin prick hyperalgesia, a dysfunction of Aβ fibres.

Inserted in between the pinprick stimuli, in order to obtain a measure of dynamic mechanical allodynia (DMA; a triggering of a pain response from stimuli which do not normally provoke pain, representing an increased response of neurons), a set of three light tactile stimulators of moving innocuous stimuli; cotton wisp exerting a force of 3mN, a q-tip exerting a force of 100mN and a standardized brush exerting a force of 200-400mN (Somedic, Sweden) were applied, each with a single stroke, 2cm in length.

A total of 50 stimuli; 15 tactile and 35 pinprick, were delivered with the participant giving numerical ratings for each stimulus. These stimuli were presented in runs of 10, pseudo random sequences, each consisting of three tactile and seven pinprick stimuli, each with a 10s interval (below the critical frequency for wind-up).

MPS was calculated as the geometric mean of all numerical ratings for pinprick stimuli, while DMA was the geometric mean of all rating for all three of the light touch stimulators.

**Wind-up ratio (WUR)**

To establish a measure of wind-up ratio, a test of temporal summation (WUR; a frequency dependent increase in excitability of spinal cord neurons), the perceived intensity of a single 256mN pinprick stimulus was compared with that of a series of 10 repetitive stimuli of the same physical pinprick intensity (256mN, 1/s applied within an area of 1cm^2^). Participants gave a numerical pain rating representing the single stimulus, and then an estimated mean over the whole series of 10, using a 0-100 numerical rating, as described above. The whole procedure was then repeated five times. The wind-up ratio was calculated as the ratio of the mean of the five series divided by the mean of the five single stimuli.

**Vibration detection threshold (VDT)**

Vibration detection threshold (VDT) was performed with a tuning fork (64Hz, 8/8 scale) placed over the bony premise of the wrist (processus styloideus ulnae). VDT was determined with three series of descending stimulus intensities; measured by the number on a scale of 8, at which the stimulus ceased to be felt (8 meaning no vibration stimuli = 0Hz). The threshold is then the mean of three stimulus repetitions and evaluates vibration sensation mediated by Aβ fibres.

**Pressure pain threshold (PPT)**

The pressure pain threshold (PPT) was performed over the thenar eminence (muscle on the palm of hand at the base of the thumb) with a handheld pressure algometer (Somedic) with a 1cm^2^ probe area. This can exert forces up to 2000kPa. The threshold was determined with three ascending stimulus intensities, each applied as a slowly increasing ramp of 50kPa/s, until participants report a painful sensation. This evaluates pressure pain sensation mediated by Aδ and C-fibres.
